# Sport engagement and life satisfaction in late childhood: a population-based analysis

**DOI:** 10.3389/fspor.2026.1784535

**Published:** 2026-05-28

**Authors:** Erik Grasaas, Øyvind Sandbakk, Christina Åsan Grasaas, Sergej M. Ostojic

**Affiliations:** 1 Teacher Education Unit, University of Agder, Kristiansand, Norway; 2 Department of Physical Performance, Norwegian School of Sport Sciences, Oslo, Norway; 3 School of Sport Science, UiT, The Artic University of Norway, Tromsø, Norway; 4 Department of Culture and Sports, Agder County Municipality, Kristiansand, Norway; 5 Faculty of Health Sciences, University of Pécs, Pécs, Hungary

**Keywords:** exercise habits, physical activity, school transport, sports engagement, well-being

## Abstract

**Background:**

While sport participation and physical activity are widely recognized as beneficial for children's well-being, limited research has examined how the frequency of sport engagement relates to life satisfaction (LS) in late childhood. This study aimed to (1) describe the frequency of sport participation, active transport, and LS among Norwegian children aged 10–12 years, and (2) assess the associations between sport participation frequency, active transport mode, and LS, with attention to gender differences.

**Methods:**

Cross-sectional data were drawn from the 2024 Norwegian Ungdata Junior Survey, comprising 46,941 children aged 10–12 years. Participants completed an electronic questionnaire during school hours. Linear regression models were used to examine associations between sport participation frequency, transport mode, and LS (measured on a 0–10 scale), adjusting for socioeconomic status, grade level, and centrality, and stratified by gender.

**Results:**

Boys reported significantly higher LS than girls (mean 8.4 vs. 7.7, *p* < 0.01). Among boys, LS increased with sport participation frequency, from 8.0 (no evenings) to 8.6 (five evenings per week). In contrast, girls' LS remained relatively stable across participation levels (7.7–7.8). Regression analysis showed the strongest positive associations with LS observed for children participating in organized sports one to three evenings per week. No significant associations were found between type of transport and LS for either gender.

**Conclusions:**

In a large sample of representative Norwegian children aged 10–12 years of age, LS scores for boys progressed by the frequency of weekly sport participation but remained stable for girls. Still, the strongest positive associations between sport participation and LS were among those engaging in sports one to three sessions per week, which suggests that a balanced approach to youth sports, emphasizing enjoyment, autonomy, and social connection, may be more effective in promoting well-being than maximizing participation frequency in late childhood.

## 1 Introduction

Physical activity (PA) is widely recognized to increase well-being and provide health-related benefits for children and adolescents (1, 2). Regular engagement in PA during childhood is associated with numerous health benefits, including improved physical fitness (3, 4), mental health (5, 6), and social development (7, 8). Conversely, physical inactivity is a major risk factor for non-communicable diseases (9–13) and has been linked to adverse health trajectories later in life (9, 10). Despite these well-documented benefits, global data indicate that the majority of children and adolescents do not meet the World Health Organization's (WHO) recommendation of at least 60 min of moderate-to-vigorous PA per day (14). Organized sport participation is often promoted as a key strategy to increase PA levels among youth, offering structured opportunities for movement, skill development, social interaction and well-being (11, 13).

Life satisfaction (LS), a core component of subjective well-being, reflects an individual's overall evaluation of their life quality (15). Among adolescents, higher levels of PA and sport participation have been consistently associated with greater LS (16–21). However, the nature of this relationship in younger children, particularly those aged 10–12 years, remains less well understood. This developmental stage is characterized by significant physical, emotional, and social changes, and is often marked by declining PA levels and increasing dropout from organized sports (22, 23). These shifts may have important implications for children's well-being, especially as they transition into adolescence.

The frequency of sport participation may relate differently to LS among boys and girls due to gendered differences in motivation and socialization processes in early adolescence. Boys are more likely to report higher levels of sport-related competence and derive satisfaction from performance, whereas girls' experiences are often more contingent on relational factors such as social support and the quality of the environment (24, 25). In addition, socialization processes shape how boys and girls experience sport. Organized sport has historically been associated with masculine norms, which may provide boys with greater opportunities for social recognition and status, whereas girls may be more exposed to evaluative pressures, including body image concerns and social comparison, which can attenuate the positive effects of participation (25, 26). Consequently, increased frequency of sport participation may translate more directly into higher LS for boys, while for girls the association may depend more on contextual factors than on frequency alone.

Moreover, the assumption that organized sport participation serves as a reliable proxy for overall PA may not hold true across all demographic groups. For instance, children from immigrant backgrounds may participate less in organized sports but remain physically active through informal or unstructured activities (27). Additionally, recent evidence suggests that the relationship between PA and LS may be non-linear, with diminishing returns or even negative associations at very high levels of activity (28). This raises questions about the optimal frequency of sport participation for promoting well-being in children.

Another factor potentially influencing LS is the mode of school transportation for children (29). Active transport, such as walking or cycling, has been linked to increased autonomy, PA, and environmental engagement in adults (30), all of which may contribute to well-being. However, findings among children are scarce and mixed, with some studies reporting unexpected negative associations between active commuting and well-being in girls (31), highlighting the need for further investigation.

Given these gaps, this study aims to extend current knowledge by examining the associations between sport participation frequency, active transport, and LS in a large, nationally representative sample of Norwegian children aged 10–12 years. Specifically, the objectives are to: (1) describe the frequency of sport participation, active transport, and LS in this population; and (2) explore the associations between sport participation frequency and active transport mode with LS, stratified by gender. Understanding these relationships may inform more effective, developmentally appropriate strategies to support children's well-being through PA.

We hypothesized that higher frequency of sport participation would more directly translate into higher LS among boys compared to girls.

## 2 Methods

### 2.1 Data collection

Ungdata Junior is a nationwide survey administered annually by Norwegian Social Research (NOVA) at Oslo Metropolitan University, in collaboration with the Regional Centre for Drug Rehabilitation (KoRus) and the Municipalities sector's organization (KS). The initiative produces national reports each year, represented by different counties. The collected data is considered representative, as data accumulated over the preceding three-year period includes all Norwegian counties. Ungdata Junior has since 2017 had more than 200.000 responders aged 10–12 years.

Municipalities coordinate with schools to facilitate participation, and the survey is administered electronically during a scheduled school hour. Pupils who do not participate in the survey are assigned alternative academic tasks by their teachers. According to Ungdata, the information gathered through these surveys serves as a valuable resource for planning and implementing interventions targeting youth welfare and public health in Norway (32). The project is financed through allocations from the Norwegian national budget via grants from the Norwegian Directorate of Health. For more information, Løvgren & Jacobsen have published a protocol for the motivation, development and use of Ungdata Junior (33).

### 2.2 Study design and participants

This current study used an observational cross-sectional study design (34), collected from the 2024 Ungdata Junior Survey. The study was conducted from February to March 2024 and includes Norwegian children aged 10–12 years. We analyzed 46,941 children from the 2024 survey wave. In 2023–24 combined, 65,252 children participated nationwide, underscoring the 2024 sample's representativeness. All data is anonymous. However, due to smaller sample sizes in distinct variables, complimentary information of participants, such as municipality affiliation is not included in this current dataset due the Ungdata Junior's privacy guidelines and general data protection rules (GDPR).

### 2.3 Study variables

The Ungdata Junior study entails all aspects of children's life combined with various health-related questions.

#### 2.3.1 Sport participation

Levels of sport participation were assessed using the question: “*Do you regularly participate in any leisure activities?*”. Respondents could choose from three response alternatives, “*Yes*”*, “No, but used to be*” and “*No, never participated in organized activities*”*.* If yes, the participants were presented with the statement “*Sports or athletics*” with a simple “*yes*” or “*no*” response option. Only respondents who answered “*yes”* to Sports/athletics were asked about weekly evenings of activity. To assess the frequency of sport participation, the following question were administered: “*From Monday to Friday, how many evenings do you participate in regular leisure activities?*”. Respondents could choose from six response alternatives, ranging from “*No evenings*”, gradually up to “*Five evenings*”.

#### 2.3.2 Active transport

Levels of active transport were assessed with the question: “*How did you get to school today?*”. Responders could choose from three alternatives: “*I walked or cycled*”, “*I was driven by car*” or “*I used the bus or other public transport*”.

#### 2.3.3 Life satisfaction

LS was assessed by using the question “*Overall, how satisfied are you with your life right now? Please rate your answer on a scale of 0–10, where 0 means you are not at all satisfied and 10 means you are very satisfied*”. The question derives from the ladder of Cantril (1965). Higher scores indicated better LS. Across populations, LS as a single-item measure has reported satisfying validity compared to multiple-item LS scale, including children and adolescents from 10 to 19 years of age (35–37).

#### 2.3.4 Covariates

Relevant covariates in regression models were socioeconomic status (SES), centrality and grade level. SES was calculated by summing four items (car ownership, child's own bedroom, holiday frequency, computer/tablet count), with higher scores indicating higher SES. Centrality (urban–rural) was included to control for locale differences in activity opportunity. Grade level used as a proxy for age and was assessed by using the question “*Which grade level are you in?”.* Responders were provided three response alternatives: “Grade level 5,” “Grade level 6” and “Grade level 7”.

### 2.4 Ethical consideration

Prior to the survey, all pupils were informed that the survey is voluntary, and they were free to skip questions if preferable. They can also withdraw any time if they wish. Two weeks in advance, parents/guardians receive written information regarding the purpose of the study and that participation is voluntary. Parents/guardians may reserve their child from participation. This procedure for recruitment is approved by the Norwegian Agency for Shared Services in Education and Research (ref. 821474), known as SIKT [*Norwegian Agency for Shared Services in Education and Research (SIKT)*, Accessed 05.10.2023], they have also approved all questions used in Ungdata. The Ungdata survey aligns with the Helsinki Declaration. To ensure a transparent and systematic reporting, this study has followed the Strengthening the Reporting of Observational Studies in Epidemiology (STROBE) guidelines (38), see Supplementary Material S1.

For most children, the questions in the survey are unproblematic to answer. Some may still find certain topics difficult. Ungdata has therefore taken measures to reduce any inconvenience that participants in Ungdata junior may experience, such as parents and pupils are informed in advance that they may contact the school health service or the Red Cross call service if they wish to speak to an adult afterwards. In addition, the school health service is instructed to be on standby in connection with the survey (39).

### 2.5 Statistical analyses

All statistical analyses were performed using IBM SPSS Statistics for Mac, Version 25.0 (IBM Corp., Armonk, NY, USA). Descriptive measures for continuous variables are reported as means and corresponding standard deviation (SD). Categorical variables are presented as counts and percentages. Independent t-test was employed to compare differences in mean for LS among boys and girls. Prior to regressions, assumption of normality was examined using standard diagnostic procedures and was sufficiently satisfied. Normality of residuals was assessed with visual inspection of histograms and Q–Q plots, as well as values of skewness and kurtosis were within ±2, which is indicative of acceptable normality. Homoscedasticity was also evaluated and did not indicate substantial heteroscedasticity. Linear regressions were conducted to explore the relationship between the frequency of sport participation (independent variables), type of transport (independent variables) and LS (dependent variable). To examine how frequency of sport participation was associated with LS across gender and on separate weekdays, while controlling for socioeconomic status (SES), centrality, and age, a linear regression model stratified by both gender and weekdays were employed. This approach allows for a more nuanced understanding of subgroup differences, while maintaining a robust analytical framework that accounts for key confounders. The predicting independent variables for type of transport were recorded into dummy variables and entered the regression model stratified by sport participation or not participating Results are presented with R^2^, coefficients, confidence intervals and *p*-values, and adjusted for SES, age (grade level) and centrality. All tests were two-sided and *p*-values of <0.05 were considered statistically significant. Given the high response rate in the presented study variables and large sample size, neither imputation nor bootstrapping was deemed necessary.

## 3 Results

### 3.1 Participants

This current study includes a total of 46,941 Norwegian children aged 10–12 years from Ungdata Junior study, with a close to equal distribution between boys and girls (50.2% vs. 49.8%, respectively) and between grade levels [5th grade (32.6%), 6th grade (33.3%) and 7th grade (34.1%)]. Most of the children (98.5%) responded whether they participated in organized activities, herein a total of 35,731 children responded that they participated in organized sports (77.3%). Please see Supplementary File S2 for an overview of response rate for study variables.

### 3.2 Descriptive statistics

LS mean score (SD) for the total sample was 7.9 (SD 2.2), with significant higher scores reported by boys than in girls (*P* value < 0.01, 8.4 vs. 7.7, respectively). Close to half of the boys (49.4%) and girls (45.8%) reported an active form of transport to school, such as walking or cycling. The majority of children (52.4%) reported transport by car, bus or other public transport while ∼48% used active transport (Table 1). The frequency of weekly sport participation was quite similar among boys and girls. Notably, 3.0% of boys and 1.4% of girls reported doing organized sports but had zero weekday sessions (perhaps indicating infrequent or weekend-only participation, or a period with illness or injury).

**Table 1:** Characteristics of study variables.

Study variable	All (*N* %)	Girls (*N* %)	Boys (*N* %)
Life satisfaction (mean/SD)	7.9 (2.2)	7.7 (2.2)	8.4 (1.8)**
Transport to school			
Walking or cycling	47.6%	45.8%	49.4%
Driven by car	29.0%	30.2%	27.9%
Bus or public transport	23.4%	23.9%	22.7%
Sport participation			
No evening sessions	2.2%	1.4%	3.0%
One evening session	11.0%	10.5%	11.5%
Two evening sessions	31.6%	31.8%	31.4%
Three evening sessions	27.8%	30.2%	25.5%
Four evening sessions	18.0%	18.4%	17.7%
Five evening sessions	9.3%	7.7%	10.9%

** *P* < 0.01

LS scores for boys progressed by the frequency of weekly sport participation (Table 2), revealing scores from 8.0 (no evenings) gradually up to 8.6 (five evenings). The same pattern was not uncovered among girls, with scores ranging from 7.7 (no evenings) to 7.8 (five evenings). The proportion of both boys and girls using active transport progressed with the frequency of weekly sports participation, indicating higher proportion of active transport among the already most active children.

**Table 2:** Life satisfaction and active transport stratified by gender and the frequency of sport participation.

Study variable	No evenings	One evening	Two evenings	Three evenings	Four evenings	Five evenings
Life satisfaction						
Girls	7.7 (2.7)	7.6 (2.4)	7.6 (2.2)	7.8 (2.1)	7.9 (2.0)	7.8 (2.2)
Boys	8.0 (2.3)	8.2 (2.0)	8.4 (1.8)	8.5 (1.7)	8.5 (1.8)	8.6 (1.9)
Active Transport						
Girls	39.5%	38.7%	43.4%	47.1%	49.2%	54.2%
Boys	46.6%	38.4%	47.0%	51.4%	54.6%	57.2%
Driven by car						
Girls	31.8%	33.0%	31.2%	29.7%	28.4%	28.2%
Boys	30.8%	32.7%	28.3%	26.6%	25.9%	26.2%
Bus or public						
Girls	24.4%	28.3%	25.4%	23.2%	22.4%	17.6%
Boys	22.6%	28.9%	24.7%	22.1%	19.5%	16.6%

**Figure 1:**
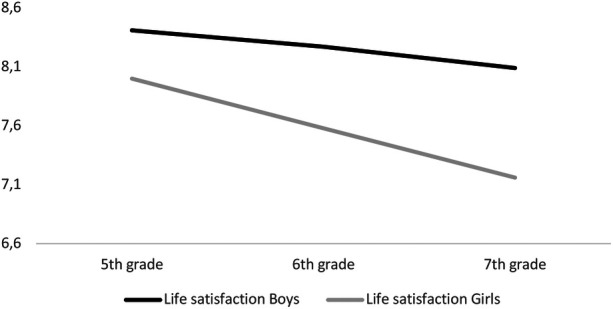
Life satisfaction across grade levels for boys and girls.

Descriptive findings revealed that LS decreased by grade levels (Figure 1). Girls' mean LS dropped from 8.0 in 5th to 7.2 in 7th grade, vs. boys' drop from 8.4 to 8.1, which may indicate that this period of time is particularly critical for girls.

### 3.3 Regressions analyses

Regressions between the frequency of sport participation and LS revealed strongest associations for boys and girls who engaged in one to three evening sessions per week (Table 3). Boys reporting sport participation five evenings per week uncovered borderline positive associations [B = 0.45; 95% CI (−0.03 to 0.93)] after adjusting for SES, grade level and centrality, whereas girls reporting sport participation five evenings per week unveiled nonsignificant findings with a negative coefficient [B = −0.23; 95% CI (−0.76 to 0.30)], which points to the necessity of limiting overly rigid training structures at this age, particularly for girls.

**Table 3:** Regressions of the frequency of sport participation (independent variables) on life satisfaction (dependent variable)..

Study variable	Boys	95% CI	*P* value	R^2^	Girls	95% CI	*P* value	R^2^
	B				B			
Sport participation								
No evenings	0.09	−0.34 to 0.52	0.69	0.01	0.29	−0.35 to 0.93	0.37	0.04
One evening	0.28	0.08 to 0.49	<0.01**	0.01	0.23	0.04 to 0.43	0.02*	0.03
Two evenings	0.39	0.18 to 0.61	<0.01**	0.01	0.15	−0.02 to 0.32	0.09	0.03
Three evenings	0.35	0.06 to 0.64	0.02*	0.01	0.28	0.04 to 0.51	0.02*	0.02
Four evenings	0.28	−0.23 to 0.79	0.28	0.01	0.02	−0.37 to 0.41	0.92	0.02
Five evenings	0.45	−0.03 to 0.93	0.07	0.02	−0.23	−0.76 to 0.30	0.39	0.03

Adjusted for socioeconomic status, grade level (age) and centrality.

* *p* < 0.05.

** *p* < 0.01.

Results from crude regressions between the type of transport (active transport, driven by car or bus/public transport) and LS revealed all nonsignificant findings (*p* = 0.45, 0.61 and 0.68, respectively). Findings remained nonsignificant after adjusting for SES, grade level and centrality and stratification of gender and sports participation (Table 4, all *p* > 0.05).

**Table 4:** Linear regression of transport mode (active vs. motorized) on life satisfaction, stratified by sport participation status.

Study variable	Boys	95% CI	*P* value	R^2^	Girls	95% CI	*P* value	R^2^
Type of transport	B				B			
Sport participants				0.01				0.02
Active transport	−0.48	−1.15 to 0.18	0.16		0.14	−0.71 to 0.98	0.75	
Driven by car	−0.23	−0.56 to 0.11	0.19		0.03	−0.39 to 0.46	0.88	
By bus or public transport	−0.18	−0.41 to 0.04	0.11		−0.04	−0.32 to 0.24	0.79	
Nonparticipants				0.02				0.01
Active transport	−0.78	−3.75 to 2.19	0.61		1.53	−0.73 to 3.78	0.19	
Driven by car	−0.49	−1.97 to 1.00	0.52		0.70	−0.43 to 1.83	0.23	
By bus or public transport	−0.31	−1.30 to 0.68	0.54		0.45	−0.31 to 1.20	0.24	

Adjusted for socioeconomic status, grade level (age) and centrality.

* *p* < 0.05.

** *p* < 0.01.

## 4 Discussion

This study explored the associations between sport participation frequency, active transport, and LS among Norwegian children aged 10–12 years. The main findings revealed that boys reported significantly higher LS than girls, and that LS increases with sport participation frequency among boys. In contrast, girls' LS remained relatively stable across participation levels. Interestingly, when controlling for several covariates, regression analysis showed the strongest positive associations with LS observed for both boys and girls participating in sports one to three evenings per week. No significant associations were found between types of transport and LS for either gender.

While specific average LS scores for children aged 10–12 using the Cantril ladder (0–10) are not comprehensively detailed across all countries, available data derived from the Health Behavior in School-aged Children (HBSC) indicate average scores around 7–7.5 among European children aged 10–12 (40), which indicate somewhat lower scores than our sample (7.9). Another notable finding is the decline in LS across grade levels, particularly among girls. This trend aligns with developmental research indicating that early adolescence is a critical period marked by increased vulnerability to declines in mental health and subjective well-being, especially for girls (41). The sharper decline observed in girls may be influenced by a combination of biological, psychological, and social factors, including the onset of puberty, increased academic and social pressures, and shifting self-perceptions.

These results contribute to a growing body of literature emphasizing the benefits of PA for youth well-being. However, the observed gender differences in LS across the frequency of sport participation indicate this is more complex than previously assumed. The diminishing or absent returns for girls at higher levels of sport participation may reflect psychosocial or contextual factors, such as differing motivations for participation, energy levels, social dynamics within sports teams, or the nature of coaching and competition environments. In addition, the tendency towards reduced LS with the highest level of sport participation among girls resonate with the “over-scheduling hypothesis”, which posits that excessive structured activity may lead to stress, reduced autonomy, or burnout, particularly in younger populations (42).

Interestingly, after adjustment for SES, age and centrality in the regression analyses, the relationship between sport participation frequency and LS was strongest with modest involvement per week for both boys and girls. This contrasts with the descriptive analyses, which suggested gender-specific patterns. The discrepancy indicates that the covariates act as confounding variables, which thereby masked the relationship in the unadjusted data, as reliance on descriptive statistics alone may lead to interpretations that are not supported when potential confounders are considered. Thus, our findings suggest a non-linear relationship between sport participation and LS in late childhood, wherein neither lower nor higher frequency of sport participation confer any additional benefits for either gender. In light of this discussion, it is crucial to continue advocate PA engagement for children aged 10–12 years, as most children worldwide do not adhere to the PA recommendations (14). However, there might be great benefits in unstructured play and multidisciplinary sports, as they foster all-round development and promote a greater versatility in children, which may hold benefits for both their well-being and long-term sport performance. Recent discoveries on the acquisition of the highest levels of human performance by Güllich and colleagues (43), uncovered that world-class performance is associated with limited discipline-specific practice in early years, but with increased multidisciplinary practice and with gradual progress.

Moreover, developing children's creativity and social-emotional competencies through play and not only through organized activities may have several various positive social behaviors outcomes, positive communication among group members, increasing assertive cognitive strategies and thereby enhancing relationship and well-being (44). In line with this, findings has suggested that children that participated in team sports or in both team and individual sports had higher well-being than children who participated in individual sports (45). In contrast, the highest LS scores among older adolescents were found in individual sports (46), indicating a shift of benefits in sports during adolescence.

The lack of significant associations between active transport and LS was not unexpected, given prior studies suggesting that active commuting can enhance mood, autonomy, and social interaction (3). One possible explanation is that the measure used in this study, mode of transport on a single day, may not capture habitual patterns or the subjective experience of commuting. For instance, previous research has highlighted that travel satisfaction, rather than mode alone, may be more predictive of well-being outcomes (36). Additionally, in a country like Norway, where active transport is relatively common and infrastructure supports walking and cycling, the variability in experience may be too limited to detect meaningful differences in LS.

### 4.1 Practical implications

Overall, these findings underscore the importance of considering both the quantity and quality of sport participation. While moderate involvement appears beneficial, excessive frequency may not yield additional advantages and could even be counterproductive for girls. This has practical implications for parents, educators, trainers, and sports organizations aiming to promote youth well-being through PA. In a family setting, parents and caregivers should give encouragement through supportive routines by focusing on the joyfulness of the sports, the benefits of hard effort and long-term investments, rather than focusing on early results and short-term wins. Further, our findings underscore the importance of predictable and balanced frameworks in learning environments. Children should be given time to develop role understanding, self-regulation, and cooperative skills, rather than being pressured toward early specialization or constant performance evaluation, as they are more likely to develop resilience and durable competence with long-term perspectives. Collective structures and long-term support are central for the facilitation of continuous well-being and success. Overall, this study provides useful insights for parents, coaches and teachers regarding the role of organized sports involvement, including the fact that there exists a saturation point for participation in early adolescence linked to wellbeing, as well as the importance of gender differences in this context. Note that our findings do not suggest that children should avoid being very active, quite the contrary; however, it seems that unstructured activities and movement that promotes enjoyment, social connectedness is pivotal, rather than early excessive pressure. Within this framework, parents, coaches and teachers should foster balanced environments between organized and unorganized activities to ensure the children's wellbeing.

### 4.2 Pedagogical implications

In Norway, as most countries in the western world, a growing polarization between physically active and inactive youth is emerging. Self-organized play is declining, and as children enter adolescence, many drop out of organized sports. We therefore suggest that schools must assume a more central role in promoting inclusive physical activity during this transitional period. Integrating activity breaks, active learning strategies, and opportunities for exploratory “sampling” of different sports within the curriculum can support the development of fundamental motor skills and movement literacy for all children and youths, regardless of socioeconomic background. At the same time, a balance between organized and unorganized sports, which includes play-based forms of activity is essential, as less structured, non-assessed movement experiences may support autonomy, enjoyment, and overall wellbeing. In addition, closer collaboration between schools and local sports organizations can create structured yet accessible arenas for participation, reducing dependence on parental resources and voluntary engagement. In practice, this may involve partnerships with local sports clubs to provide after-school programs on school premises, the involvement of coaches in physical education or sports clubs, and the use of school facilities for community-based activities outside school hours. Importantly, such initiatives should emphasize participation, enjoyment, and mastery rather than competition or assessment, as performance pressure is often cited as a key factor in children's and adolescents' disengagement from organized sport. Offering diverse, non-competitive and socially oriented activities, ranging from traditional team sports to dance or outdoor pursuits, can foster a sense of belonging and increase overall participation. These school–sport collaborations may also help lower practical barriers such as cost and transportation, thereby enhancing inclusivity. In this way, the school structure emerges as a pedagogical key site for democratizing access to physical activity, addressing structural inequalities, and compensating for the limitations of the voluntary organized sports model by reaching students who might otherwise remain inactive. The question is if today's politicians are willing to do such an investment for wellbeing and health in future generations?.

### 4.3 Strengths and limitations

Several key factors have underscored this study's credibility. The use of a large population-based sample among children aged 10–12 with a high response rate, should be considered a strength. The Ungdata Junior (for children) follows the same rigor procedures set by the original Ungdata study (for adolescents) for cleaning data and the omission of corrupt data (32). Further, using LS as an indicator for subjective well-being in children, is a reliable and valid assessment tool, which allows for easy interpretation across ages, cultures and countries, which increases the external validity of the findings. Moreover, the study benefits from following the STROBE guidelines, which ensures a systematic and transparent form of reporting.

Our study also has some limitations warrant for consideration. First, all data analysed were cross-sectional, which precludes the identification of any causal relationships. Second, our findings may not be generalized to other populations in other countries. Third, due to the large number of questions in Ungdata Junior, many of the single-item questions are not validated. Fourth, sport participation may not be a completely reliable proxy for PA among children (27), however due to GDPR restrictions and privacy rules, we are unable to address potential covariates such as ethnicity or other relevant demographic variables. Fifth, despite 97.2% of the participating children responded that they reported honest answers, only 84.2% found the questions easy to answer, which might indicate a petite risk of recall biases or interpretations challenges in children aged 10–12 years. Finally, the frequency of sport participation reported here does not reflect the total weekly sport participation, as weekend activity is not included. This should be considered as a limitation. Furthermore, a single-day report of transport mode may introduce measurement error and potential misclassification bias, as it may not accurately capture habitual behaviour and can be influenced by day-to-day variability, such as weather conditions or atypical schedules.

### 4.4 Future directions

To build upon these findings, future research should consider the following avenues:

1. Future studies should differentiate between types of sports (e.g., team vs. individual, competitive vs. recreational) and consider contextual factors such as coaching style, peer relationships, and parental involvement. These variables may moderate the impact of sport participation on LS.
2. Regarding active transport, incorporating measures of travel satisfaction, perceived safety, and social support during commuting could offer a more nuanced understanding of how school travel influences well-being.
3. Expanding the scope to include other forms of PA, such as unstructured play, household chores, or digital fitness engagement, may provide a more holistic view of children's activity patterns and their relationship with LS.
4. iv. Longitudinal studies are needed to establish causal relationships between sport participation and LS. Tracking children over time would help determine whether changes in sport involvement precede changes in well-being or vice versa.
5. Qualitative research could provide deeper insights into the lived experiences of children, particularly girls, who engage in high-frequency sports. Understanding their motivations, perceived pressures, and social dynamics could illuminate why increased participation does not translate into higher LS for this group.
6. Finally, cross-cultural comparisons could help determine whether these findings are specific to the Norwegian context or generalizable to other countries with different cultural, infrastructural, and educational systems.

## 5 Conclusion

In a large sample of representative Norwegian children aged 10–12 years of age, LS scores for boys progressed by the frequency of weekly sport participation but remained stable for girls. This study highlights the nuanced relationship between sport participation and LS in late childhood. While moderate engagement in organized sports is associated with higher LS, excessive participation does not appear to confer additional benefits. These findings suggest that a balanced approach to youth sports, emphasizing enjoyment, autonomy, and social connection, may be more effective in promoting well-being than maximizing participation frequency in late childhood.

Moreover, the absence of a significant association between school transport mode and LS indicates that other factors, such as the quality of the commuting experience or broader lifestyle variables, may play a more critical role in shaping children's subjective well-being. As children transition into adolescence, particularly girls may face increasing challenges with their well-being. Interventions aimed at sustaining or enhancing LS during this period should consider gender-specific needs, the structure of activities, and the broader psychosocial environment. By tailoring strategies to the developmental and contextual realities of children's lives, stakeholders can better support their health, happiness, and long-term engagement in PA.

## Data Availability

Publicly available datasets were analyzed in this study. This data can be found here: Data supporting the results of this study is available upon request from the Norwegian Agency for Shared Services in Education and Research (SIKT) [47]. Reference to dataset from SIKT: NOVA and Bakken, Anders. (2024). Ungdata 2010-2023 [https://doi.org/10.18712/NSD-NSD3157-V1].

## References

[1] Kumar B Robinson R Till S. Physical activity and health in adolescence. Clin Med (Lond). (2015) 15(3):267–72. 10.7861/clinmedicine.15-3-267 26031978 PMC4953112

[2] Organization, W. H. Global Recommendations on Physical Activity for Health (2010). 26180873

[3] Kriemler S Meyer U Martin E van Sluijs EM Andersen LB Martin BW. Effect of school-based interventions on physical activity and fitness in children and adolescents: a review of reviews and systematic update. Br J Sports Med. (2011) 45(11):923–30. 10.1136/bjsports-2011-090186 21836176 PMC3841814

[4] Poitras VJ Gray CE Borghese MM Carson V Chaput JP Janssen I Systematic review of the relationships between objectively measured physical activity and health indicators in school-aged children and youth. Appl Physiol Nutr Metab. (2016) 41(6 Suppl 3):S197–239. 10.1139/apnm-2015-0663 27306431

[5] Biddle S Atkin A Cavill N Foster C. Correlates of physical activity in youth: a review of quantitative systematic reviews. Int Rev Sport Exerc Psychol. (2011) 4(1):25–49. 10.1080/1750984x.2010.548528

[6] Booth JN Ness AR Joinson C Tomporowski PD Boyle JME Leary SD Associations between physical activity and mental health and behaviour in early adolescence. Ment Health Phys Act. (2023) 24:100497. 10.1016/j.mhpa.2022.100497

[7] Burdette HL Whitaker RC. Resurrecting free play in young children: looking beyond fitness and fatness to attention, affiliation, and affect. Arch Pediatr Adolesc Med. (2005) 159(1):46–50. 10.1001/archpedi.159.1.46 15630057

[8] Mendonça G Cheng LA Mélo EN de Farias Júnior JC. Physical activity and social support in adolescents: a systematic review. Health Educ Res. (2014) 29(5):822–39. 10.1093/her/cyu017 24812148

[9] Barnekow-Bergkvist M Hedberg G Janlert U Jansson E. Prediction of physical fitness and physical activity level in adulthood by physical performance and physical activity in adolescence—an 18-year follow-up study. Scand J Med Sci Sports. (1998a) 8(5):299–308. 10.1111/j.1600-0838.1998.tb00486.x 9809389

[10] Glenmark B Hedberg G Jansson E. Prediction of physical activity level in adulthood by physical characteristics, physical performance and physical activity in adolescence: an 11-year follow-up study. Eur J Appl Physiol Occup Physiol. (1994a) 69(6):530–8. 10.1007/BF00239871 7713074

[11] Granger E Di Nardo F Harrison A Patterson L Holmes R Verma A. A systematic review of the relationship of physical activity and health status in adolescents. Eur J Public Health. (2017a) 27(suppl_2):100–6. 10.1093/eurpub/ckw187 28340201

[12] Guinhouya BC Samouda H de Beaufort C. Level of physical activity among children and adolescents in Europe: a review of physical activity assessed objectively by accelerometry. Public Health. (2013) 127(4):301–11. 10.1016/j.puhe.2013.01.020 23582270

[13] Hallal PC Victora CG Azevedo MR Wells JC. Adolescent physical activity and health: a systematic review. Sports Med. (2006) 36(12):1019–30. 10.2165/00007256-200636120-00003 17123326

[14] Guthold R Stevens GA Riley LM Bull FC. Global trends in insufficient physical activity among adolescents: a pooled analysis of 298 population-based surveys with 1·6 million participants. Lancet Child Adolesc Health. (2020) 4(1):23–35. 10.1016/s2352-4642(19)30323-2 31761562 PMC6919336

[15] Diener E. Subjective well-being. Psychol Bull. (1984) 95(3):542–75. 6399758

[16] Feng B Xu K Zhou P. Association between vigorous physical activity and life satisfaction in adolescents. Front Public Health. (2022) 10:3–4. 10.3389/fpubh.2022.944620 PMC9607945 36311584

[17] Gao J Nie Y Guo M Tang W Qu G Wang X Analysis of the association between adolescent physical activity and life satisfaction: a systematic review and meta-analysis. BMC Psychol. (2025) 13(1):738. 10.1186/s40359-025-02847-1 40616133 PMC12228328

[18] Grasaas E Ostojic S Sandbakk Ø. Associations between levels of physical activity and satisfaction with life among Norwegian adolescents: a cross-sectional study. Front Sports Act Living. (2024) 6:3–6. 10.3389/fspor.2024.1437747 PMC11324472 39149573

[19] Riddervold S Haug E Kristensen SM. Sports participation, body appreciation and life satisfaction in Norwegian adolescents: a moderated mediation analysis. Scand J Public Health. (2024) 52(6):704–10. 10.1177/14034948231184525 37403364

[20] Urchaga JD Guevara RM Cabaco AS Moral-García JE. Life satisfaction, physical activity and quality of life associated with the health of school-age adolescents. Sustainability. (2020) 12(22):9486. 10.3390/su12229486

[21] Zullig KJ White RJ. Physical activity, life satisfaction, and self-rated health of middle school students. Appl Res Qual Life. (2011) 6(3):277–89. 10.1007/s11482-010-9129-z

[22] Crane J Temple V. A systematic review of dropout from organized sport among children and youth. Eur Phy Educ Rev. (2015) 21(1):114–31. 10.1177/1356336(14555294

[23] Fraser-Thomas J Côté J Deakin J. Examining adolescent sport dropout and prolonged engagement from a developmental perspective. J Appl Sport Psychol. (2008) 20(3):318–33. 10.1080/10413200802163549

[24] Fredricks JA Eccles JS. Family socialization, gender, and sport motivation and involvement. J Sport Exerc Psychol. (2005) 27(1):3–31. 10.1123/jsep.27.1.3

[25] Slater A Tiggemann M. Uncool to do sport”: a focus group study of adolescent girls’ reasons for withdrawing from physical activity. Psychol Sport Exerc. (2010) 11(6):619–26. 10.1016/j.psychsport.2010.07.006

[26] Holt NL Neely KC Slater LG Camiré M Côté J Fraser-Thomas J A grounded theory of positive youth development through sport based on results from a qualitative meta-study. Int Rev Sport Exerc Psychol. (2017) 10(1):1–49. 10.1080/1750984X.2016.1180704 27695511 PMC5020349

[27] Nielsen G Hermansen B Bugge A Dencker M Andersen LB. Daily physical activity and sports participation among children from ethnic minorities in Denmark. Eur J Sport Sci. (2013) 13(3):321–31. 10.1080/17461391.2011.635697 23679149

[28] Mutz M Reimers AK Demetriou Y. Leisure time sports activities and life satisfaction: deeper insights based on a representative survey from Germany. Appl Res Qual Life. (2021) 16(5):2155–71. 10.1007/s11482-020-09866-7

[29] Waygood EOD Friman M Taniguchi A Olsson LE. Children’s life satisfaction and travel satisfaction: evidence from Canada, Japan, and Sweden. Travel Behaviour Soc. (2019) 16:214–23. 10.1016/j.tbs.2018.04.004

[30] Ettema D Friman M Gärling T Olsson LE. Travel mode use, travel mode shift and subjective well-being: overview of theories, empirical findings and policy implications. In: Wang D He S, editors. Mobility, Sociability and Well-being of Urban Living. Springer Berlin Heidelberg (2016). p. 129–50. 10.1007/978-3-662-48184-4_7

[31] Tsiros MD Samaras MG Coates AM Olds T. Use-of-time and health-related quality of life in 10- to 13-year-old children: not all screen time or physical activity minutes are the same. Qual Life Res. (2017) 26(11):3119–29. 10.1007/s11136-017-1639-9 28674767

[32] Frøyland LR. Ungdata—lokale Ungdomsundersøkelser. Dokumentasjon av Variablene I Spørreskjemaet. Oslo: NOVA (2017). p. 13–7.

[33] Løvgren M Jacobsen SE. Motivation, development and use of Ungdata Junior: an annual general survey among children in Norway. Scand J Public Health. (2024) 52(4):521–7. 10.1177/14034948221150038 36803224

[34] O'Donoghue P. Research Methods for Sports Performance Analysis. 1st ed. New York: Routledge (2009). 10.4324/9780203878309

[35] Cheung F Lucas RE. Assessing the validity of single-item life satisfaction measures: results from three large samples. Qual Life Res. (2014) 23(10):2809–18. 10.1007/s11136-014-0726-4 24890827 PMC4221492

[36] Jovanović V. The validity of the satisfaction with life scale in adolescents and a comparison with single-item life satisfaction measures: a preliminary study. Qual Life Res. (2016) 25(12):3173–80. 10.1007/s11136-016-1331-5 27262574

[37] Lukoševičiūtė J Gariepy G Mabelis J Gaspar T Joffė-Luinienė R Šmigelskas K. Single-item happiness measure features adequate validity among adolescents. Front Psychol. (2022) 13:4–5. 10.3389/fpsyg.2022.884520 PMC9274985 35837634

[38] von Elm E Altman DG Egger M Pocock SJ Gøtzsche PC Vandenbroucke JP. The strengthening the reporting of observational studies in epidemiology (STROBE) statement: guidelines for reporting observational studies. Ann Intern Med. (2007) 147(8):573–7. 10.7326/0003-4819-147-8-200710160-00010 17938396

[39] Ungdata Junior. Available online at: https://www.ungdata.no/hva-er-ungdata-junior/ (Accessed November 7, 2025) (2025).

[40] Hodačová L Hlaváčková E Sigmundová D Kalman M Kopčáková J. Trends in life satisfaction and self-rated health in Czech school-aged children: HBSC study. Cent Eur J Public Health. (2017) 25:S51–6. 10.21101/cejph.a4820 28752749

[41] Daly M. Cross-national and longitudinal evidence for a rapid decline in life satisfaction in adolescence. J Adolesc. (2022) 94(3):422–34. 10.1002/jad.12037 35390206

[42] Mahoney JL Harris AL Eccles JS. Organized activity participation, positive youth development, and the over-scheduling hypothesis. Social Policy Rep. (2006) 20(4):1–32. 10.1002/j.2379-3988.2006.tb00049.x

[43] Güllich A Barth M Hambrick DZ Macnamara BN. Recent discoveries on the acquisition of the highest levels of human performance. Science. (2025) 390(6779):eadt7790. 10.1126/science.adt7790 41411418

[44] Garaigordobil M Berrueco L Celume MP. Developing children’s creativity and social-emotional competencies through play: summary of twenty years of findings of the evidence-based interventions “game program”. J Intell. (2022) 10(4). 10.3390/jintelligence10040077 36278599 PMC9590021

[45] Vella SA Cliff DP Magee CA Okely AD. Sports participation and parent-reported health-related quality of life in children: longitudinal associations. J Pediatr. (2014) 164(6):1469–74. 10.1016/j.jpeds.2014.01.071 24657117

[46] Grasaas E Sandbakk Ø. Life satisfaction across sports disciplines and sports categories among Norwegian adolescents: comparisons to national data. Front Psychol. (2025) 16. 10.3389/fpsyg.2025.1577326 40547591 PMC12179080

[47] SIKT. Norwegian Agency for Shared Services in Education and Research (SIKT). Available online at: https://sikt.no/en/home (Accessed 5 October, 2023).

